# Supplementary material for: The genome sequence of the European ground squirrel, Spermophilus citellus (Linnaeus, 1766)
Source: Wellcome Open Res. 2025 Apr 8;10:184. [Version 1] doi: 10.12688/wellcomeopenres.23974.1 (PMC12107237; doi:10.12688/wellcomeopenres.23974.1)
Supplement: Peer review history

# Reviewer report: Reviewer response for version 1

**DOI:** 10.21956/wellcomeopenres.26450.r122130

**Published:** 2025-05-26

**Authors:** Pawel Michalak

**Affiliations:** 1 Edward Via College of Osteopathic Medicine (Ringgold ID: 41066), Monroe, Louisiana, USA; 2 University of Haifa, Haifa, Israel

This Data Note describes the chromosome-level genome assembly of the European ground squirrel, an endangered rodent species endemic to Central and Southeastern Europe. Using Pacific Biosciences HiFi long-read sequencing and Hi-C scaffolding, the authors report a high-quality genome assembly of 3.09 Gb, with 95.48% of the sequence scaffolded into 20 chromosomal pseudomolecules including the X chromosome. The mitochondrial genome was also assembled. The authors provide extensive details on sample collection, sequencing protocols, assembly pipelines, and quality assessment metrics, including QV scores, BUSCO completeness (96.1%), and k-mer completeness (99.41%). The study aims to contribute to the genomic resources of S. citellus to support research in evolutionary biology, population genetics, and conservation genomics, especially given the species' status as endangered and its ecological importance.

 This is a well-executed and clearly presented genome assembly report. The data quality is high, the methodological framework is sound, and the dataset is well positioned to benefit researchers in genomics, ecology, and conservation biology. The only minor recommendation I would offer is to expand the background to reference existing studies on comparative genomics across rodents, chromosomal polymorphisms or karyotypic evolution in Spermophilus and related taxa, which would strengthen the evolutionary rationale for the genome assembly.

 Incidentally, I couldn’t help but notice that one of the reviewers expressed concern about the use of GenomeScope2 with PacBio data, suggesting the software is not appropriate for high-error-rate reads. However, the authors used PacBio HiFi sequencing, which achieves an accuracy of 99.9% (0.1% error rate), comparable to Illumina. GenomeScope2 is widely and successfully used with HiFi data, as demonstrated in recent studies (e.g., https://www.nature.com/articles/s41597-024-03808-w).

Are sufficient details of methods and materials provided to allow replication by others?

Yes

Is the rationale for creating the dataset(s) clearly described?

Partly

Are the datasets clearly presented in a useable and accessible format?

Yes

Are the protocols appropriate and is the work technically sound?

Yes

Reviewer Expertise:

Genomics, evolution, bioinformatics

I confirm that I have read this submission and believe that I have an appropriate level of expertise to confirm that it is of an acceptable scientific standard.

## References

[1] : First genome assembly of the order Strepsiptera using PacBio HiFi reads reveals a miniature genome. *Sci Data* .2024;11(1) : 10.1038/s41597-024-03808-w 934 10.1038/s41597-024-03808-w 39198488 PMC11358474

# Reviewer report: Reviewer response for version 1

**DOI:** 10.21956/wellcomeopenres.26450.r122136

**Published:** 2025-04-24

**Authors:** Gerrit Wehrenberg

**Affiliations:** 1 University of Oulu, Oulu, Finland

I want to thank the authors for their work and the submission of this article.

 The Data Note titled ‘ *The genome sequence of the European ground squirrel, Spermophilus citellus (Linnaeus, 1766)*’ by Dimitra-Lida Rammou, Dionisios Youlatos, and Alexandros Triantafyllidis provide and present the chromosome-level genome assembly and its methodological pipeline of two female European ground squirrels ( *Spermophilus citellus*) from a population in Thessaloniki, Greece.

 The authors provide a solid background on the focal species of the study and clearly explain the sequencing project within which the presented genome assembly was generated.

 I think the Background section would be more valuable with mentioning the evolutionary research done in this clade, since a chromosome-level genome assembly could play an important role in this field. There are for example interesting evolutionary karyotype patterns in the Nearctic and Palearctic realms. Genomic data could shed a new light on this matter.

 The validation of the karyotype in line with earlier studies would strengthen your findings as well. Generally, the authors should point out more why this novel genome assembly is important overall and specifically for this species/higher taxon with referencing literature and maybe its conservation situation.

 In the framework of a data note the workflow protocol was overall well provided.

 Please consider to not utilise *GenomeScope2* for PacBio reads since this software is not fully appropriate for data with high sequencing error rates (e.g., PacBio reads). *GenomeScope2* is designed for accurate *k*-mer profiles, typically from Illumina reads. There are alternatives providing estimates of genome size, heterozygosity, and repeat content more suitable for your data.

 Utilised field work, laboratory methodology and software (including versions) are provided. The provision of detailed information on the type locality and the studied population is particularly commendable, as such data are crucial for the prospective application of the genome assembly in population genetic research.

 Please provide the information of the morphological sex identification method of the live specimens. Especially, since often very small Y-gonosomes can be hard to detect in Hi-C data even with a coverage of 30x. This would strengthen your molecular findings.

 Specimen and sequencing data as well as assembly statistics are provided and the presented genome assembly is already assessable publicly.

 I approve this article for publication to the editor, provided that the minor revisions mentioned above are addressed.

Are sufficient details of methods and materials provided to allow replication by others?

Yes

Is the rationale for creating the dataset(s) clearly described?

Yes

Are the datasets clearly presented in a useable and accessible format?

Yes

Are the protocols appropriate and is the work technically sound?

Partly

Reviewer Expertise:

Wildlife and Conservation genomics

I confirm that I have read this submission and believe that I have an appropriate level of expertise to confirm that it is of an acceptable scientific standard, however I have significant reservations, as outlined above.

# Reviewer report: Reviewer response for version 1

**DOI:** 10.21956/wellcomeopenres.26450.r122129

**Published:** 2025-04-18

**Authors:** Camila do Nascimento Moreira

**Affiliations:** 1 Universidade Federal de Mato Grosso Do Sul, Campo Grande, Brazil

The article brings new sequencing data about the European squirrel *Spermophilus citellus*, a rodent species classified as endangered. The sequencing process and assembly were so well-detailed. Also, all data produced and software used are available to be checked. However, I have some suggestions to be incorporated into the manuscript, as follows:

 1. The species full name must be cited only once, upcoming citations must abbreviate the genus name;

 2. Specify the meaning of the abbreviation ‘EU’ in the third sentence of the second paragraph of the section ‘Background’. Also, the meaning of ‘.io’ at the end of the second sentence of the ‘Nucleic acid extraction’ section;

 3. I suggest changing the section from ‘Nucleic acid extraction’ to ‘DNA extraction’, since only DNA was sequenced in the work;

 4. Finally, I suggest giving more attention to the chromosome composition of the species. Since details about the species taxonomy, morphology, ecology, and distribution were so well presented. What is the morphology of the chromosomes of this species? Is there any polymorphism reported for this species? At least for the X chromosome the answer is yes, see: Chassovnikarova T, et al., 2015 (Ref 1)

Are sufficient details of methods and materials provided to allow replication by others?

Yes

Is the rationale for creating the dataset(s) clearly described?

Yes

Are the datasets clearly presented in a useable and accessible format?

Yes

Are the protocols appropriate and is the work technically sound?

Yes

Reviewer Expertise:

Genome evolution, chromosome rearrangements, repetitive DNA

I confirm that I have read this submission and believe that I have an appropriate level of expertise to confirm that it is of an acceptable scientific standard.

## References

[1] : Sex chromosome variability of Spermophilus citellus (Linnaeus, 1766) in the Southeastern part of the Balkan Peninsula. *Mammalian Biology* .2015;80(4) : 10.1016/j.mambio.2014.10.002 365-371 10.1016/j.mambio.2014.10.002
